# Associations Between Diet, Oral Health, and General Development in Romanian School-Age Children

**DOI:** 10.3390/nu17172832

**Published:** 2025-08-30

**Authors:** Ana-Gabriela Seni, Andreea Sălcudean, Ramona-Amina Popovici, Dora-Mihaela Cîmpian, Teodora Olariu, Iustin Olariu, Mariana Păcurar, Andreea Mihaela Kiș, Silviu-Constantin Bădoiu, Viorel Jinga, Alexandru Blidaru, Silviu-Ionel Dumitrescu, Ramona-Camelia Anculia, Norina Forna, Liana Todor, Monica Tarcea

**Affiliations:** 1 Doctoral School of Faculty of Medicine, “George Emil Palade” University of Medicine, Pharmacy, Science and Technology of Târgu Mureș, 540139 Târgu Mureș, Romania; gabriela.seni@umfst.ro; 2 Department of Ethics and Social Sciences, “George Emil Palade” University of Medicine, Pharmacy, Science and Technology of Târgu Mureș, 540139 Târgu Mureș, Romania; dora.cimpian@umfst.ro; 3 Department of Management and Communication in Dental Medicine, Department I, Faculty of Dental Medicine, “Victor Babes” University of Medicine and Pharmacy of Timisoara, 300041 Timisoara, Romania; 4 Department of Emergency, “Vasile Goldis” Western University of Arad, 310025 Arad, Romania; olariu.teodora@uvvg.ro; 5 Department of Dentistry, Faculty of Dental Medicine, “Vasile Goldis” Western University of Arad, 310414 Arad, Romania; olariu.iustin@uvvg.ro; 6 Department of Orthodontics, Faculty of Dental Medicine, “George Emil Palade” University of Medicine, Pharmacy, Science and Technology of Târgu Mureș, 540139 Târgu Mureș, Romania; marianapac@yahoo.com; 7 Research Center for Pharmaco-Toxicological Evaluations, Faculty of Pharmacy, “Victor Babes” University of Medicine and Pharmacy, Eftimie Murgu Sq., No. 2, 300041 Timisoara, Romania; kis.andreea@umft.ro; 8 Faculty of Medicine, University of Medicine and Pharmacy “Carol Davila” Bucharest, 020021 Bucharest, Romania; silviu.badoiu@umfcd.ro (S.-C.B.); viorel.jinga@umfcd.ro (V.J.); alexandru.blidaru@umfcd.ro (A.B.); 9 Department of Cardiology I, Dr. Carol Davila Central Military Emergency Hospital, 010825 Bucharest, Romania; silviu.dumitrescu@scumc.ro; 10 Department of Cardiology, Faculty of Medicine, Titu Maiorescu University, 040051 Bucharest, Romania; 11 Discipline of Occupational Medicine, Faculty of Medicine, “Victor Babes” University of Medicine and Pharmacy of Timisoara, 300041 Timisoara, Romania; ramona.anculia@umft.ro; 12 Implantology and Prosthetic Implant Rehabilitation Department, Faculty of Dental Medicine, “Grigore T. Popa” University of Medicine and Pharmacy, 700115 Iasi, Romania; norina.forna@umfiasi.ro; 13 Department of Dental Medicine, Faculty of Medicine and Pharmacy, University of Oradea, 10 Decembrie Sq., 410068 Oradea, Romania; liana.todor@gmail.com; 14 Department of Community Nutrition, Faculty of Medicine, “George Emil Palade” University of Medicine, Pharmacy, Science and Technology of Târgu Mureș, 540139 Târgu Mureș, Romania; monica.tarcea@umfst.ro

**Keywords:** dental caries, oral health behaviors, dietary patterns, body fat, child health

## Abstract

Background: The prevalence of dental caries has increased among children, largely due to nutritional habits or inadequate access to dental care. The present study aimed to investigate the prevalence of dental caries associated with various factors that lead to their appearance, such as food intake and body composition, among Romanian school-age children from two counties, Bistriţa Năsăud and Mureş. Methods: This cross-sectional study included 1100 children aged 6–10 years from two Romanian counties. Dental caries experience was assessed using the DMFT and dmft indices based on WHO criteria. Dietary intake and oral health behaviors were evaluated through a food frequency questionnaire completed by parents. Associations between dietary variables and dental caries were assessed using independent sample *t*-tests and Mann–Whitney U tests. A binary logistic regression model was used to estimate the likelihood of caries in the permanent dentition (DMFT > 0), with covariates including county, parental education, daily sugar intake, consumption of dairy products, body mass index (BMI), and waist-to-height ratio. Results: It was found that the children who daily consume cheese recorded a DMFT value lower than the children who did not consume (*p* < 0.05). Moreover, those who consume sugary foods recorded higher values of DMFT as compared with those who did not (*p* < 0.05). The body weight, BMI, and waist circumference are positively correlated with DMFT, but negatively with dmft (*p* < 0.05). The overall prevalence of caries was 79.8% in primary dentition and 63.6% in permanent dentition, with slightly higher rates observed in Bistriţa-Năsăud County compared to Mureş. It seems that the predictors of the caries’s presence among children include the mother’s education level, sugar intake frequency, and body fat ratio. Conclusions: Based on significant associations found, one can affirm that the dental caries of the Romanian school-age children is due to sugary food intake and body composition. In addition, the direct relationship between dental caries and childhood obesity showed through the correlation of BMI and dental health indices denotes that school-age children should reduce sugary foods and increase dairy products.

## 1. Introduction

The phase of a child’s development towards adolescence is characterized by significant physiological, psychological, and social changes. At the same time, this period is also marked by changes in nutritional habits and oral health behaviors, which will influence long-term health outcomes. From a nutritional point of view, adolescence involves increased dietary requirements due to rapid growth and biological changes. Nutritional choices made during this period can have lasting health implications, influencing the likelihood of developing conditions such as obesity and diabetes later in life [1,2]. Therefore, the need for balanced nutrition is essential at this transitional stage, since insufficient intake may lead to growth delays and health complications [3,4]. Adopting healthy eating habits early on supports a child’s immediate growth needs and establishes patterns that continue into adulthood. However, socio-economic status and environmental factors often impede access to diverse and nutritious food choices, particularly in low-resource settings [5].

The relationship between dietary intake and oral health is a substantial area of study that highlights how dietary habits can affect oral health status, and, conversely, how oral health can influence dietary choices. Adequate nutrition is vital for the development and maintenance of oral structures, while deficiencies of essential nutrients can lead to various oral health problems, including tooth decay, periodontal disease, and tooth loss. Certain nutrients play a constructive role in maintaining oral health and preventing disease. For example, antioxidant vitamins and omega-3 polyunsaturated fatty acids have been associated with a decreased progression of periodontal disease among adults, suggesting a direct impact of dietary choices on oral health outcomes [6]. Furthermore, periodontal disease and tooth loss can lead to chewing difficulties, restricting food choices, and leading to nutritional deficiencies [7,8,9,10,11]. Reduced ability to consume fibrous and nutrient-rich foods, due to missing teeth or periodontal disease, compromises the overall nutritional status of the individual [12,13]. Therefore, nutritional awareness can significantly influence the oral health and dietary practices of adults, who will understand that education plays a key role in promoting better health outcomes for their children [14]. Poor dietary patterns, characterized by high consumption of sugary and highly processed foods, significantly correlate with higher incidences of dental caries and other oral health issues in children. It has been stated that dietary habits play a crucial role in overall health status and oral health, establishing a link between high cariogenic food intake and an increased risk of dental caries among school children [15]. Similarly, Carvalho Silva et al. demonstrated a direct relationship between children’s dietary patterns and the development of dental problems, highlighting the critical role that nutrition plays in maintaining oral health [16]. The quality of diet not only affects oral health but is also intertwined with systemic health outcomes. Common risk factors for overweight and obesity, prevalent in children, include poor dietary choices that are linked to dental caries [17]. Unhealthy dietary patterns have been identified as both direct causes of tooth decay and contributors to obesity, which can exacerbate health issues such as diabetes and cardiovascular diseases later in life [18,19]. The presence of dental caries can lead to significant functional limitations, which adversely affect quality of life and can result in decreased nutritional intake due to pain or discomfort while eating [20].

The relationship between tooth decay and consumption of sugary foods is well established, and the strong association between high sugar intake and increased prevalence of tooth decay has often been demonstrated [21,22,23,24]. Tooth decay is a multifactorial disease that develops mainly due to the consumption of sugar, which creates an acidic environment in the mouth, facilitating the demineralization of tooth enamel. Confounding factors such as poor eating habits contribute significantly to the risk of tooth decay. For example, skipping meals and daily consumption of sugar-based beverages have been identified as risk factors, indicating a clear link between unhealthy eating practices and the increased prevalence of tooth decay [25].

Compared to occasional or consistently low sugar intake, the frequency of sugar intake also plays a key role. Based on a longitudinal study, it was shown that children with increasing trajectories of sugar intake had a significantly higher prevalence of cavities compared to those with a consistently low sugar intake [26].

The World Health Organization (WHO) reports a widespread prevalence of tooth decay among children, with estimates suggesting that between 60% and 90% may be affected globally, making tooth decay a major public health crisis [27,28]. Epidemiologic studies indicate a prevalence of dental caries among children aged 6–10 years ranging from 43% to over 90% worldwide [29,30]. Among the key factors contributing to this prevalence are eating habits (especially excessive consumption of sugary snacks and drinks), oral hygiene practices, socioeconomic status, and access to dental care [31,32]. A high percentage of children do not have proper brushing routines or access to preventive dental services. Shaffer et al. found that socio-economic disadvantage correlates directly with higher rates of caries, particularly in children from poorly developed communities [33]. This is consistent with findings indicating that children from low-income families have a higher prevalence of untreated dental caries due to financial constraints and limited access to dental care [34]. Therefore, addressing this public health problem requires comprehensive strategies that include oral hygiene education, improved access to dental care, and public health interventions focusing on dietary changes to reduce sugar consumption among children.

In Romania, there is a worryingly high prevalence of dental caries in children, with significant associations reported between caries prevalence and dietary habits, especially high sugar consumption among children aged 6–12 years, emphasizing the importance of nutrition in dental health outcomes [35]. The DMFT index (decayed, missing, and filled teeth), which is a standard measure for dental caries, is particularly high in this age cohort. It has been shown that mean DMFT values increased substantially as children moved towards adolescence, with peak values observed around the age of 9 years [36]. This observation is consistent with findings indicating that caries experience increases with age, attributed primarily to prolonged exposure to cariogenic factors [30]. It has been reported that up to 63.2% of children between the ages of 6 and 10 may suffer from dental caries, highlighting the urgent need for effective preventive strategies to combat the problem globally [37].

The existing literature highlights the significant influence of dietary habits, socioeconomic factors, and oral hygiene practices on the prevalence of dental caries globally and in various populations. However, there is a notable lack of comprehensive data specifically examining the interplay between food intake, body composition, and dental caries among school-aged children in Romania, particularly in different regional contexts. This gap leaves a limited understanding of how local dietary patterns and socio-economic disparities contribute to caries prevalence within this population. Therefore, the present study aims to investigate the prevalence of dental caries in Romanian school-age children from the counties of Bistriţa Năsăud (northern Romania) and Mureş (north-central Romania), focusing on how food consumption and body composition influence caries development. The Bistriţa Năsăud county is divided administratively into a municipality (Bistrița, which is the county seat), 3 towns (Năsăud, Beclean, Sângeorz-Băi), and 59 communes. In 2021, the county had a total population of 295,988 inhabitants. On the other hand, the Mureş county has 102 administrative-territorial units: 4 municipalities (among which Târgu Mureș is the county seat), 7 cities, and 91 communes. In 2011, the county had a total population of 550,846 inhabitants. This research seeks to fill the knowledge gap by providing region-specific insights to guide targeted preventive strategies.

## 2. Materials and Methods

### 2.1. Participants, Study Design, and Ethical Considerations

The present cross-sectional study comprised a randomized sample of school-age children, evaluated using primary data collected from a total of 1100 participants, consisting of primary school children aged 6 (with mixed dentition) and 10 years (with permanent dentition). The study employed a two-stage random sampling design. In the first stage, public schools from two Romanian counties (Bistriţa-Năsăud and Mureş) were stratified by urban and rural location based on official Ministry of Education records. The schools from urban areas were randomly selected using a computer-generated random number list to ensure geographic and demographic representation. In the second stage, within each selected school, one class from each target age group (6 and 10 years old) was randomly chosen using simple random sampling based on class rosters provided by school administrators. All children in the selected classes who met the inclusion criteria (enrolled full-time, with parental consent, and no diagnosed systemic illness) were invited to participate. No cluster sampling weights were applied. The overall process aimed to reduce selection bias and ensure that the final sample was representative of the target population within the selected counties.

The children, together with their parents, were invited to participate in the study. The inclusion criteria consisted of children belonging to the age groups of 6 and 10 years. The children who suffer from mental or physical disease were excluded, since the associated disease may influence the dental caries and dietary intake. The second excluded criterion was related to the children’s age (in the study, only children aged 6 and 10 years were included). From a dental perspective, this age group typically experiences the transition from primary (deciduous) teeth to permanent teeth. Studies on dental age estimation reveal that the maturation of teeth during this phase is crucial for determining biological age and assessing nutritional status, as well as potential developmental health issues [38,39]. In addition, the interaction between dental development and broader health indicators emphasizes the importance of this age group. Malnutrition and socioeconomic factors may negatively affect dental development more visibly in this age group compared to older children [40].

Before the dental clinical screening and completing the questionnaire, parents and their children were informed about the content of the study and the steps that must be taken to complete it. Thus, written informed consent was obtained from both parents and a verbal consent from the children.

Based on the written informed consent given by the parents, the study received the approval of the Research Ethics Committee of UMFST Târgu Mureș (Approval Code: 3147, approval Date: 20 May 2024). The primary school authorities of the four schools in urban areas of the Bistriţa Năsăud and Mureş counties also granted permission to conduct the study. To ensure confidentiality, each child was recorded under a unique numerical code.

Specialists from the “George Emil Palade” University of Medicine, Pharmacy, Science and Technology from Târgu Mureş visited the four schools in each county to collect data over 3 months (September 2024–November 2024) regarding the questionnaire.

Regarding the dental clinical screening of the children, this was performed by pediatric dentists from the Faculty of Dental Medicine, “George Emil Palade” University of Medicine, Pharmacy, Science and Technology from Târgu Mureș, Romania. The pediatric dentists used dental mirrors, tongue depressors, and a WHO periodontal probe, working under a reflector light. The gauze and toothpicks were also used to clean teeth and remove food remnants, ensuring the screening’s consistency and efficiency.

### 2.2. Data Collection

The information about the children’s sugar intake (consumption of sweets, cookies, and chocolate), and information about the children’s daily consumption of dairy products (consumption of milk, yogurt, and cheese), were collected through a food frequency survey, and the parent educational level (primary, secondary, or higher) was self-reported. When it was necessary, the dietary intake (dairy products and sugary foods) was completed by asking each child also about the food and beverages consumed the day before. Details of food preparation methods and ingredients used, including the trade name of the products, were also recorded. The amount of food consumed the previous day was reported in terms of standardized containers (bowl, cup, glass, and spoon). The dietary intake (dairy products and sugary foods) frequency was categorized as 1–2 times a day, 1–2 times a week, 3 times a week, 4–5 times a week, 1–2 times a month, and never, over 3 months.

The oral health behaviors included tooth-brushing frequency (ranging from once a day to irregular), using fluoride toothpaste, as well as the frequency of dentist visits (ranging from once a year to when they have a complaint). The dental clinical screening followed the WHO recommendations regarding the oral health surveys [41]. A cavitated lesion was considered dental caries. Data collected included the number of healthy teeth, decayed teeth, filled teeth, and missing teeth. From the total number of decayed, missing, and filled teeth (DMFT and dmft), one could assess the dental caries of the primary and permanent teeth, according to WHO guidelines [41]. The decayed, missing, and filled teeth (DMFT) index, together with its counterpart for primary teeth (dmft), is a fundamental epidemiological tool for assessing dental health among primary school children. The DMFT index quantifies the prevalence of dental caries by assessing the total number of decayed, missing, and filled permanent teeth, and the dmft index refers to primary teeth, allowing the assessment of dental caries affecting children before the transition to permanent dentition. We considered the prevalence of caries as the proportion of children with caries classified as affected (DMFT/dmft > 0) and children with caries classified as unaffected (DMFT/dmft = 0).

Children were measured regarding their body height, waist, and hip circumferences with an accuracy of 0.1 cm, using a stadiometer as well as a nonelastic tape. Half of the distance between the lower part of the rib cage and the upper part of the iliac crest was considered the waist circumference, and the widest part of the buttocks was considered the hip circumference [42]. The body mass index (BMI) values were calculated according to the following formula:

(1) $$BMI\left[\frac{kg}{m^{2}}\right]=\frac{bodyweight[kg]}{{height}^{2}[m^{2}]}$$

According to WHO (2007) reference values, the BMI children classification was according to BMI for age [%], as follows: (1) underweight (<5%); (2) healthy weight (5–85%); (3) overweight (85–95%); (4) obese (≥95%) [43]. The children’s body fat was assessed by the bioelectrical impedance analysis method by two nutritionist doctors.

To ensure data accuracy and methodological rigor, specific attention was given to the validation and reliability of the instruments used. The food frequency questionnaire (FFQ) employed in this study was adapted from previously validated dietary assessment tools used in pediatric populations and reviewed by a panel of experts in nutrition and pediatric dentistry to ensure content validity. A pilot study involving 30 children was conducted before the main data collection phase to assess clarity, comprehension, and response variability, resulting in minor refinements of wording and structure. Although no formal psychometric validation (e.g., test–retest reliability or construct validity analysis) was conducted due to resource limitations, the questionnaire showed consistent response patterns and acceptable feasibility in the pilot sample. Regarding the clinical dental assessments, all oral examinations were performed by experienced pediatric dentists (2 teams formed by a pediatric dentist and a hygienist) who were trained in the WHO oral health survey criteria [44]. Before fieldwork, a calibration session was conducted to minimize inter-examiner variability. Although Kappa statistics were not formally computed, periodic consensus discussions were held during the data collection period to ensure diagnostic consistency, particularly in borderline cases of cavitated lesions. This approach enhanced the reliability of the caries detection protocol and reduced examiner subjectivity.

### 2.3. Statistical Analysis

By using the IBM-SPSS software platform (from IBM^®^ SPSS^®^ Statistics, software version 24.0), the statistical analysis was performed. By applying the Kolmogorov–Smirnov test, the quantitative data were evaluated, with normal or non-normal distribution. The data were expressed as mean ± standard deviation (SD) in the case of normally distributed variables. For non-normal distributed variables, the data were expressed as medians and interquartile ranges. The nominal variables were expressed as numbers (*n*) and percentages (%). To compare categorical data, the Fisher chi-square test was used. The correlation between quantitative variables was assessed either by Pearson correlation coefficient (when at least one variable showed normal distribution) or by Spearman correlation coefficient (when neither variable showed normal distribution). To estimate the probability of having caries in the permanent dentition, a binary logistic regression model was applied. The following independent variables were considered: parents’ education level (self-reported), frequency of tooth brushing, frequency of sugary foods intake, and the child’s body fat value. According to the DMFT values, the caries were classified as present or absent, using an interval of 95% confidence.

Regarding data completeness, the dataset was reviewed before analysis to identify any missing values. The overall rate of missing data was low (<5%), and no variable critical to the primary outcomes (e.g., DMFT/dmft scores, age, sex, sugar intake frequency, or parental education) had more than 2% missing observations. For descriptive analyses and bivariate comparisons, complete-case analysis was performed. In the case of multivariate modeling (logistic regression), records with missing values in the included predictors were excluded using listwise deletion. Given the low proportion of missingness and the assumed missing-at-random (MAR) mechanism, no imputation methods were applied. This conservative approach preserved the internal validity of the models without introducing bias due to data manipulation.

In addition to *p*-values, 95% confidence intervals (CIs) were calculated and reported for all mean comparisons and effect estimates to improve the interpretability of the results. For continuous variables (e.g., DMFT/dmft indices), independent sample *t*-tests were applied, and CIs were computed for the mean differences between groups (e.g., daily consumers vs. non-consumers of dairy or sugary products). These CIs provide a range within which the true population mean difference is expected to lie with 95% certainty, thereby offering insight into the magnitude and direction of observed effects.

In the case of non-normally distributed variables, median differences were reported, and the Mann–Whitney U test was used; however, due to the limitations of this test in producing interpretable effect sizes, CIs were not computed for these comparisons. For logistic regression analyses, adjusted odds ratios (AORs) were presented alongside their respective 95% CIs to quantify the strength and precision of associations between predictors and caries prevalence. Reporting confidence intervals along with *p*-values enhances the robustness of the statistical analysis by avoiding overreliance on arbitrary significance thresholds and allows for better understanding of both clinical and statistical relevance.

Given the number of statistical comparisons performed in the analysis (e.g., multiple group comparisons across food categories and counties), the potential for increased type I error was considered. However, no formal adjustment for multiple comparisons was applied. This decision was based on the exploratory nature of the analysis and the primary aim to identify associations rather than to test a priori hypotheses. Instead, interpretation of *p*-values was made with caution, particularly for marginally significant results (e.g., *p*-values close to 0.05), and supported by effect size estimates and CIs. We acknowledge that the absence of correction may increase the likelihood of false-positive findings and recommend that results be interpreted in the context of the broader pattern of associations observed.

## 3. Results

Among the 1100 Romanian school-age children screened, the proportion of counties was similar (550 children/county) as well as the proportion of children school age and boys and girls (50.5% vs. 49.5% for Bistriţa-Năsăud County and 50.2% vs. 49.8% for Mureş County). In regard to the education level, only 150 mothers of Bistriţa-Năsăud children had a higher education level as compared with 284 mothers of Mureş children. In both groups of children, the percentage of mothers with higher education is higher than that of fathers. In regard to the employment status, one can observe that in Bistriţa-Năsăud County, 34.9% of mothers were unemployed and 25.8% of fathers vs. 29.6% of unemployed mothers and 24.2% of fathers in Mureş County (Table 1).

Table 2 presents the oral health behaviors of Romanian school-age children by the county from which they originate. One can observe that there was no statistically significant difference (*p* = 0.177, *p* > 0.05) between the children from both counties as regards the brushing frequency, because, of all the school-age children (*n* = 1100), only 43.4% brushed their teeth once a day, 30.9% brushed twice a day, and 25.7% declared that they brushed their teeth irregularly. The answers regarding the use of fluoride toothpaste were also statistically insignificant when the Pearson chi-square test was applied (*p* = 0.608; *p* > 0.05).

As regards the frequency of dentist visits, most of the children from both counties (86.2%) declared that they visit the dentist only when they have a dental problem, as compared with 3.8% of children that reported once in a year visit, or with 10.0% of children who reported a twice in a year dentist visit. Regarding this question, a statistically significant difference (*p* = 0.005, *p* < 0.05) was obtained.

Table 3 depicts the mean values of dental health indicators (DMFT and dmft) for the school-age children from each county (*n* = 550) regarding the daily consumption of dairy products (milk, yogurt, cheese, and other dairy products, such as butter, sour cream, buttermilk, and mozzarella). One can observe that, in regard to the children who originate from Mureş county consuming cheese daily, it was obtained a lower value of the DMFT index as compared with the children who did not (*p* = 0.036; *p* < 0.05). Concerning the rest of the categories of daily consumption, no statistically significant differences (*p* > 0.05) were obtained between the school-age children from both counties.

Table 4 presents the mean values of dental health indicators (DMFT and dmft) according to the daily consumption of sugary foods and sugar. It can be observed that the DMFT value of the Bistriţa-Năsăud children (*p* = 0.009) and dmft value of the Mureş children (*p* = 0.028) were found to be higher in children who consumed cookies daily (*p* < 0.05).

In addition, in the case of the children originating from Mureş County, a higher DMFT value (*p* = 0.009) was obtained, concerning the daily consumption of sweets, as compared with the children who did not (*p* < 0.05). In both groups of children, higher values of DMFT were obtained regarding children who daily consumed chocolate (*p* = 0.011—in the case of Bistriţa-Năsăud children, and *p* = 0.007—in the case of Mureş children) as compared with the children who did not (*p* < 0.05).

Alongside *p*-values, all comparisons of group means are accompanied by 95% CIs to provide better estimates of effect size and assess the practical relevance of the observed differences. Including CIs in the analysis offers a more comprehensive view of data variability and clinical significance than *p*-values alone. While *p*-values indicate whether an effect exists, CIs indicate how large that effect is likely to be. In this study, the inclusion helps interpret associations such as those between dietary habits and DMFT/dmft indices more accurately, particularly in borderline significance cases.

**Figure 1:**
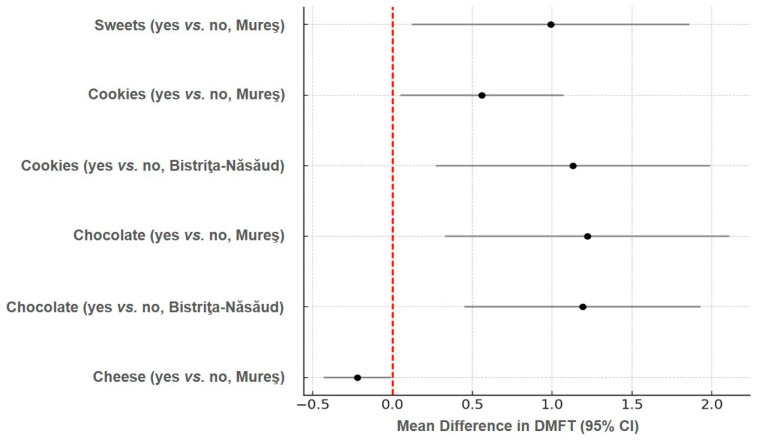
Forest Plot—Effect of selected dietary factors on DMFT scores among Romanian school-age children.

Figure 1 presents the forest plot of selected dietary predictors and their effect on DMFT scores, expressed as mean differences with 95% CIs. Chocolate and cookie consumption were associated with significantly higher DMFT values, while cheese intake showed a slight protective effect in Mureș county.

The forest plot illustrates the mean differences in DMFT scores between children who consumed specific food categories (e.g., cheese, chocolate, cookies, and sweets) versus those who did not. Each point represents the estimated mean difference, and the horizontal bars represent the 95% CIs. A positive mean difference indicates higher DMFT values in the exposed group, whereas a negative value suggests a potentially protective association. Intervals that do not cross the null value (vertical red line at 0) denote statistically significant differences. The data are stratified by county (Bistriţa-Năsăud and Mureş) and reflect results from independent sample *t*-tests. All values are rounded to two decimal places and based on sample sizes of *n* = 275 per group.

To better understand the burden of dental caries in the studied population, the overall prevalence of caries was calculated separately for the two age groups and counties, using the WHO-recommended formula detailed below, and the results obtained are presented in Table 5.

(2) $$Cariesprevalence\left[\%\right]=\frac{NumberofchildrenwithDMFTordmft>0}{Totalnumberofchildren}\times 100$$

As shown in Table 5, the prevalence of caries in primary dentition (dmft > 0) was 81.5% in children aged 6 years from Bistriţa-Năsăud County and 78.2% in those from Mureş County. In the case of children aged 10 years, who were evaluated for permanent dentition (DMFT > 0), the prevalence of dental caries was 66.5% in Bistriţa-Năsăud and 60.7% in Mureş County. These high values underscore the need for strengthened oral health strategies and public health interventions aimed at both age groups.

Table 6 presents the correlation between the anthropometric measurements (body composition) and the dental health indicators (DMFT and dmft). The results showed that in both groups of screened children, the children’s height as well as is body weight are correlated with both DMFT-dmft indices.

As regards the children’s waist circumference, this characteristic is found to be positively correlated with the DMFT values both for the group of children from Bistriţa-Năsăud (*p* < 0.01) and for the group of children from Mureş (*p* < 0.05) but negatively correlated with the dmft value (*p* < 0.05). The waist-to-hip ratio exhibits a positive correlation only with the DMFT value of the Mureş children (*p* < 0.05). The BMI characteristic showed a positive correlation with the DMFT values for both groups of children (Bistriţa-Năsăud and Mureş), and a negative correlation only with the dmft value of the Bistriţa-Năsăud children (*p* < 0.05).

Further, it analyzed the factors that are associated with dental caries, using logistic regression (presented in Table 7). Since the two counties are neighboring, and overall, the factors associated with the occurrence of dental caries are the same regardless of the origin of the study cohort, we developed a logistic regression model to predict dental caries in school-age children, aged 6–10, originating from Bistriţa-Năsăud and Mureş counties. In our model of prediction, the following associated factors were included: parents’ education level, frequency of brushing, frequency of sugar intake, and children’s body fat ratio. Our results showed that, after the logistic regression, the statistically significant variables (*p* < 0.2) found were the parents’ education level, frequency of sugar intake, as well as children’s body fat ratio. Between the two covariates concerning the parents’ education level, the mother’s education level seems to be the most significantly associated with the caries presence, alongside the frequency of sugar intake and children’s body fat ratio (*p* < 0.05), in the multiple logistic regression model.

According to the results presented in Table 7, one can observe that the children with mothers who have only primary school as an education level were 2.69 times more likely to develop dental caries than children who had mothers with a higher level of education (AOR = 2.69; 95% CI 0.82–9.06). Moreover, children who had a daily sugar intake were 1.72 times more likely to develop dental caries (AOR = 1.72; 95% CI 0.85–3.53). Finally, children who had a higher body fat ratio were also much more likely to develop dental caries in permanent dentition (AOR = 1.17; 95% CI 1.09–1.27).

## 4. Discussion

In the present cross-sectional study, we aimed to investigate the effects of dietary behaviors, as well as body composition, on the occurrence of dental caries among the Romanian school-age children from two counties. Children of 6 years (mixed dentition) and of 10 years (permanent dentition) from Bistriţa-Năsăud and Mureş counties were included in the present study. The results showed that the oral assessment and dental status of primary school children about nutrition reveal significant associations between eating habits, oral health, and general well-being. We have shown that less than half of the children assessed do not brush their teeth at least once a day. Regarding the use of fluoride toothpaste, the majority of children said that they use it, but comparing the two counties, 74.2% of children in Mureș County (which is a larger and more developed county than Bistrița-Năsăud, and most parents are highly educated) use a fluoride toothpaste. In terms of the gender of the children assessed, no difference was found. Consequently, we can state that children who have created a daily routine regarding oral hygiene (brushing their teeth at least once a day) are more likely to continue this behavior into adolescence and adulthood stages [45,46,47,48].

The use of DMFT/dmft indices in different populations, especially children, is of particular importance as they provide a clearer picture of oral health status and caries prevalence. Studies conducted in several regions of the world have widely used the DMFT index, confirming its validity in assessing caries occurrence, despite the introduction of alternative systems such as the International Caries Detection and Assessment System (ICDAS) [49,50]. In this study, DMFT/dmft indices were also used, as the WHO supports their use for the collection of robust epidemiologic data on dental caries [49,51]. In our sample, we found that 79.8% of the children aged 6 years had at least one carious, missing, or filled primary tooth (dmft > 0), while 63.6% of the children aged 10 years presented at least one affected permanent tooth (DMFT > 0). These prevalence rates are concerning and clearly exceed the WHO oral health target, which recommends that at least 50% of children aged 12 should be caries-free. Notably, caries prevalence was slightly higher in Bistriţa-Năsăud County across both age groups, which may be attributed to differences in parental education and urban development, as previously noted. Our findings align with global patterns observed in low- and middle-income countries, where caries prevalence remains elevated due to limited access to preventive care and dietary factors [52].

Varying the DMFT/dmft means has been reported among children in different regions [53,54,55]. For example, a study in Libya reported an average DMFT index of 0.86 among primary school children, in line with the WHO’s 2020 oral health targets, which recommend aiming for a DMFT of no more than 1.5 [56]. In the present study, we obtained a mean DMFT value equal to 2.02 ± 1.89 for all the Romanian schoolchildren screened. Variations in DMFT/dmft scores are often correlated with sociodemographic factors such as socio-economic status, which may have a significant impact on children’s oral health outcomes [57,58]. The dmft index was highlighted in the context of primary teeth, where the prevalence of caries is significant. Therefore, this trend underlines the need for effective preventive measures targeting early childhood [59]. Various factors influence caries prevalence and consequently DMFT/dmft scores, including oral hygiene habits, eating patterns, and parental attitudes towards dental care [60,61].

Although dairy products are often characterized by their low cariogenic potential, research has shown that they may play a beneficial role in caries prevention through their cariostatic properties, mainly due to compounds such as calcium, casein, and phosphate, which are found in milk [62,63]. The potential cariostatic properties of dairy products are mediated through a combination of nutritional and physicochemical mechanisms. Milk and dairy products are therefore less likely to contribute to tooth decay than other carbohydrate-rich foods. This may be due to, for example, the composition of milk, which is high in water and protein relative to carbohydrates. In addition, the sugars present in milk, primarily lactose, are less likely to cause tooth decay due to their slower rate of fermentation by oral bacteria. Calcium, casein, and phosphate in milk can help to remineralize enamel, an essential process to counteract the early stages of carious lesions [64]. Calcium and phosphate ions are essential in maintaining the integrity of tooth enamel, helping to neutralize acids produced by bacterial metabolism, thus preventing demineralization. Milk-derived casein phosphopeptides (CPPs), particularly found in cheese, can stabilize calcium and phosphate, increasing their availability for remineralization processes. These peptides also form a protective protein film on the tooth surface, reducing bacterial adherence and acid attack [65,66]. This mechanism highlights the potential of milk and dairy products to actively contribute to dental health by reducing the risk of caries formation [64,67,68,69]. Moreover, dairy products have a natural buffering capacity that helps neutralize acids produced by cariogenic bacteria such as *Streptococcus mutans*. This pH-modulating effect may explain why consumption of cheese after carbohydrate-rich meals is often associated with a reduction in caries risk [64]. Recent evidence also suggests that certain components in fermented dairy products, such as lactoferrin, may exhibit mild antibacterial activity, further contributing to the oral health benefits of dairy intake [70]. These mechanisms are consistent with epidemiological studies reporting inverse associations between dairy consumption and caries incidence in children and adolescents [71]. Therefore, the observed protective association of cheese intake in our study, particularly in the Mureş County subgroup, is biologically plausible and supported by both nutritional biochemistry and clinical data.

It was found that the DMFT value was lower in children who daily consumed milk than in those who did not, but without statistical significance. According to our study, Raj et al. found an inverse correlation of the whole milk intake with dental caries and BMI, among children of 6–10 years, but without statistical significance [72]. It has been reported that milk intake shows caries preventive effect but only among children (aged 6–11 years) with a high frequency of sucrose consumption [73]. Moreover, we found that the DMFT value was higher in children who eat sugary foods (sweets and chocolate), as compared with those who did not. In this context, it has been shown that the daily intake of cookies in school-age children was associated with the prevalence of caries [74]. In addition, the consumption of sugary foods among children of 6–11 years represents a predictor of dental caries. In this study, it was also shown that the daily consumption of sugar represents a predictor of dental caries (1.72; 95% CI 0.85–3.53). According to many cross-sectional studies, we also noticed a positive association between the BMI and DMFT index in children with permanent dentition [53,75,76,77,78], although some studies report that dental caries were more common in underweight children [75,79,80,81,82,83,84], or that they are associated with both high and low BMI [76].

The relationship between body mass index (BMI) and dental caries in children remains complex and multifactorial, with studies reporting positive, negative, or null associations. Several mechanisms may help explain these conflicting findings. On one hand, children with higher BMI may be more likely to consume cariogenic foods and beverages with high sugar content, which simultaneously increases the risk of both obesity and dental caries. In such cases, shared dietary risk factors, such as frequent snacking and sweetened drink intake, could explain a positive association between BMI and caries prevalence [72]. On the other hand, some studies have reported lower caries levels in overweight children, which may reflect dietary patterns high in refined carbohydrates but with reduced acidogenic exposure due to altered meal timing or lower oral retention time [85]. Additionally, parental concern over obesity might lead to better supervision of diet and oral hygiene practices, thus reducing caries risk [86]. Furthermore, variations in salivary composition, enamel maturation, or systemic inflammatory markers associated with obesity could biologically modulate susceptibility to caries, although evidence remains inconclusive [87]. Differences in study design, age groups, socioeconomic status, and access to dental care also contribute to inconsistent findings across populations [88]. These factors may partially explain the lack of significant associations observed in our study between BMI and caries experience. Future longitudinal research should aim to disentangle these mechanisms and assess causality over time.

The association between dental caries and body composition (body fat ratio and anthropometric measurements) was also assessed in the present study. The results showed a positive association between the body fat ratio and permanent teeth indicators (children of age 10), and a negative association between the body fat ratio and primary teeth indicators (children of age 6), but without statistical significance. In contrast, a study reports statistical significance when the body fat ratio was negatively correlated with dmft value and positively correlated with DMFT value [84]. The binary logistic model applied in the present study showed that in permanent dentition, the children with a higher body fat ratio are prone to developing dental caries (AOR 1.17; 95% CI 1.09–1.27). Another parameter considered an accurate marker of obesity in children is the waist circumference [89], which was positively correlated with DMFT and dmft. Our findings are in agreement with the findings reported by Peng et al. [90,91], who affirm that dental caries are associated with the central adipose layer in children aged 5 and 12 years. Several studies provide compelling evidence for the interconnections between these variables, indicating that higher sugar intake is correlated with increased BMI and body fat, which in turn may be linked to dental health problems. A positive association between obesity and dental caries among children has been reported, concluding that diets high in sugar contribute significantly to both overweight and the development of dental problems [17]. Furthermore, the authors observed that children who are overweight tend to consume large amounts of cariogenic foods—those rich in sugar—further exacerbating their risk of tooth decay [17]. Therefore, consumption of sugary foods leads to weight gain, and therefore to an increase in the incidence of dental caries, indicating that obese children are at increased risk of dental health problems compared to their non-obese counterparts [92]. But, in depth, the relationship between BMI and actual body fat in children remains complex. Although BMI is a commonly used measure, it may not always accurately reflect the degree of body fat in young populations. The interaction between body weight and dental health is underscored by additional findings that attribute similar lifestyle habits to both obesity (characterized by high sugar intake and poor food choices) and dental caries [93]. This reinforces the hypothesis that high sugar consumption may not just be an indirect indicator of higher body fat levels, but a direct factor contributing to both conditions.

While the present study explored potential associations between body composition indicators (BMI and waist-to-height ratio) and dental caries prevalence, it is important to acknowledge that some of these associations did not reach statistical significance. For instance, although children with higher waist-to-height ratio or BMI values appeared to have marginally elevated DMFT or dmft scores, the observed differences were not statistically robust (*p* > 0.05) and should be interpreted with caution. This lack of significance may be due to several factors, including limited variability in anthropometric measures within the study population, insufficient statistical power for detecting small effects, or the presence of unmeasured confounders such as dietary quality, physical activity, or metabolic status. The absence of statistically significant associations does not necessarily indicate the absence of a true relationship but rather reflects uncertainty in the current data. Future studies with larger and more diverse samples, along with longitudinal designs capable of capturing body composition dynamics over time, are warranted to better elucidate the potential interplay between childhood obesity and dental caries development. Until then, the non-significant findings in this study should be regarded as a limitation in the strength of the conclusions regarding this association.

Family education and school environment directly affect both nutritional and oral health outcomes [94,95,96]. Parents have a huge impact on the child’s education and harmonious upbringing. In Romania, it is known that the rules of conduct (the 7 years at home) are learned in the family; therefore, the child will learn correct social behavior. Subsequently, school and other educational environments confirm and reinforce the child’s family norms. Therefore, dental care habits also start at home with parents, especially mothers [97,98,99,100,101,102]. In this study, it was found that children who had a mother with a higher level of education were less prone to developing dental caries. Once again, our results are in agreement with the literature, in which it has been stated that the mother’s education level is a key factor that contributes to the odds of dental caries development [97,99,103,104]. In the multivariate logistic regression model, the mother’s level of education was identified as a significant predictor of caries presence in permanent dentition. However, the 95% CI for this variable was notably wide (AOR = 2.73, 95% CI: 0.82–9.06), indicating substantial uncertainty in the estimated effect size. This wide interval may reflect an imbalanced distribution of cases across education categories or a limited number of events within specific subgroups (e.g., mothers with higher education). Given that the total sample size was sufficient (*n* = 550 for 10-year-olds), the imprecision is more likely attributable to unequal subgroup sizes rather than an underpowered model. Furthermore, some degree of variability is expected when using categorical socioeconomic indicators in observational studies. Despite this limitation, the direction and magnitude of the association support the inclusion of maternal education in the model, and the finding aligns with existing literature linking lower parental education to increased caries risk in children [105]. Future studies may benefit from stratified sampling or larger subgroup sizes to enhance the precision of such estimates.

Overall, a poor nutritional intake is closely correlated with a decline in oral health, thus highlighting the need for integration and health promotion strategies that address both nutrition and dental hygiene in school settings. Nutrition education programs in schools, at least those in Romania, have shown promise not only in increasing children’s nutritional knowledge but also in promoting proper oral hygiene practices, reducing the prevalence of dental diseases such as caries and gingivitis. In addition, incorporating assessments of children’s eating patterns will provide specialists with essential information on their health status. Therefore, for primary school children, a holistic approach to both nutritional and dental health needs is required, which validates knowledge, involvement, and interventions that are appropriate and tailored to their unique stage of development. In Romania, the socio-economic status of families significantly influences children’s oral health status. It has been stated that, higher parental education levels correlate with improved dietary habits and lower rates of dental caries among children [105,106,107]. Therefore, it is essential for public health officials to implement educational programs aimed at increasing parental awareness of the importance of nutrition and oral hygiene. These programs could include workshops or school-based sessions to educate both parents and children about healthy eating practices and effective oral hygiene techniques [108]. Moreover, findings demonstrate a significant association between high sugar consumption and poor oral health outcomes among Romanian children [109,110]. Therefore, public health policies should encourage dietary modifications by promoting the consumption of healthier food options within schools, along with eliminating sugary snacks from school cafeterias. Collaborative initiatives with food suppliers to reduce the availability of sugary drinks and promote fruits and vegetables would also be invaluable [111]. Additionally, implementing school-based oral health programs that include regular dental check-ups can greatly enhance preventive care and heighten awareness regarding the implications of diet on oral health [112]. Such programs should integrate oral health education into school curricula and employ interactive methods to engage students effectively. For instance, using visual aids and participatory activities can enhance children’s understanding of oral hygiene and dietary practices [113]. Further, addressing the systemic barriers that hinder access to dental care is paramount. A notable proportion of children in Romania remain untreated for dental issues, emphasizing the need for accessible dental health services [112,114]. Initiatives like community health programs that offer free or subsidized dental services could serve to mitigate these gaps. Collaboration between schools, health ministries, and local dental practices may facilitate better outreach and service availability.

A key limitation of the present study lies in its cross-sectional design, which restricts the ability to establish temporal or causal relationships between dietary habits, oral health behaviors, and dental caries outcomes. As data on exposure (e.g., sugar intake, dairy consumption) and outcome (DMFT/dmft scores) were collected simultaneously, it is not possible to determine whether the observed associations reflect a true causal pathway or are influenced by unmeasured confounding factors or reverse causation. For example, children with high caries levels might have already modified their diets or oral hygiene practices at the time of assessment. The observed associations may therefore be influenced by reverse causality or residual confounding. Potential biases may have affected data collection and interpretation. Dietary information was collected using a parent-completed food frequency questionnaire (FFQ), which may be affected by both recall error and social desirability bias. Parents may underreport the frequency of cariogenic food and beverage consumption (e.g., sweets, sugary drinks) or over-report the intake of foods perceived as healthy (e.g., dairy products), especially when aware that the study concerns oral health. This type of misclassification can attenuate observed associations between diet and caries or even lead to non-differential bias, reducing the study’s ability to detect true effects. Moreover, dietary behaviors in children can be highly variable and context-dependent, and a single FFQ may not fully capture day-to-day variation or seasonal changes in intake. While the use of a standardized and previously used FFQ improves comparability, future studies should consider combining subjective reports with objective measures (e.g., 24 h recalls, biomarkers, or direct observation) to strengthen dietary exposure assessment. Moreover, the lack of formal test–retest reliability assessment for the questionnaire and the non-blinded nature of the clinical examiners may introduce measurement variability. Some degree of social desirability bias may also have influenced responses regarding oral hygiene and parental involvement. While the overall sample size was adequate, subgroup analyses (e.g., by county or education level) may have been underpowered, as reflected in some wide confidence intervals. This limits the precision of some estimates and may obscure true associations. Finally, due to the absence of follow-up, we could not assess the stability of dietary or hygiene behaviors, nor could we observe longitudinal trends in caries development across developmental stages.

Additionally, the lack of longitudinal follow-up precludes the ability to assess changes in behaviors or caries progression over time, particularly during key developmental periods such as the transition from mixed to permanent dentition. Without repeated measures, we cannot examine trajectories of risk or protection, nor can we confirm the durability of observed effects such as the cariostatic potential of dairy products or the impact of parental education.

Despite these limitations, cross-sectional studies remain valuable for generating hypotheses, identifying associations, and informing public health priorities. The large, representative sample and standardized clinical assessments used in this study enhance the internal validity of the findings, and future research efforts may benefit from cohort designs to validate and expand upon these results.

These limitations highlight the importance of future longitudinal and cohort-based studies that can better capture temporal dynamics, behavioral trajectories, and causal pathways. Such designs would allow for more rigorous testing of dietary and socioeconomic influences on oral health and provide stronger evidence for preventive strategies in pediatric populations.

## 5. Future Directions of Research

Based on the findings and limitations of this study, several recommendations for future research can be outlined:

- First, longitudinal studies are needed to establish temporal relationships between dietary patterns, oral health behaviors, body composition, and the development of dental caries. Such designs would allow for a more accurate evaluation of causality and behavioral change over time, particularly during critical periods of dentition transition.
- Second, future research should aim to improve the precision of dietary intake assessment through multi-method approaches, such as combining food frequency questionnaires with 24 h dietary recalls or objective biomarkers. This would reduce the impact of reporting bias and enhance the validity of nutritional exposure measurements.
- Third, larger and more diverse samples are recommended to increase the statistical power of subgroup analyses and to better capture variability in socioeconomic and parental factors. In particular, stratified recruitment strategies may help clarify the role of maternal education, body weight status, and access to dental care in caries risk.
- Lastly, intervention studies targeting modifiable risk factors (e.g., sugar intake, oral hygiene habits, dairy consumption) would be valuable in assessing the effectiveness of prevention strategies tailored to specific populations, especially in regions with high caries prevalence.

## 6. Conclusions

Dental caries among children aged 6–10 years in Romania remains a significant public health concern, influenced by a combination of dietary, behavioral, and socio-economic factors. Our findings suggest that higher sugar consumption and increased body fat are associated with a greater risk of dental decay in this population. Additionally, socio-demographic factors such as parental education levels appear to play an important role.

However, it is important to recognize that, due to the cross-sectional design of this study, causality cannot be established. While the observed associations provide valuable insights into potential risk factors, longitudinal research is needed to better understand the temporal relationships and causal pathways involved. Future studies incorporating prospective methods could help validate these findings and inform more targeted prevention strategies.

## Figures and Tables

**Table 1:** Demographic and socioeconomic characteristics of participating children by county.

Characteristics	Bistriţa-Năsăud County, *n* (%) (*n* = 550)	Mureş County, *n* (%) (*n* = 550)
Sex		
Male	278 (50.5)	276 (50.2)
Female	272 (49.5)	274 (49.8)
Age		
6 years	275 (50)	275 (50)
10 years	275 (50)	275 (50)
Mother’s educational level		
Primary level	221 (40.2)	98 (17.9)
Secondary level	179 (32.5)	168 (30.5)
Higher level	150 (27.3)	284 (51.6)
Father’s educational level		
Primary level	234 (42.5)	87 (15.8)
Secondary level	195 (35.5)	202 (36.7)
Higher level	121 (22.0)	261 (47.5)
Mother’s employment status		
Employed	358 (65.1)	387 (70.4)
Unemployed	192 (34.9)	163 (29.6)
Father’s employment status		
Employed	408 (74.2)	417 (75.8)
Unemployed	142 (25.8)	133 (24.2)

**Table 2:** Oral health behaviors of Romanian school-age children by the county from which they originate.

Oral Health Behaviors	Children from Bistriţa-Năsăud County (*n* = 550)		Children from Mureş County (*n* = 550)		Total of School-Age Children (*n* = 1100)		*p*
	n	%	n	%	n	%	
Tooth brushing frequency							
Once/day	262	47.6	220	40.0	477	43.4	0.177 ^a^
Twice/day	131	23.8	194	35.2	340	30.9	
Irregular	157	28.6	136	24.7	283	25.7	
Using fluoride toothpaste							
Yes	387	70.4	408	74.2	795	72.3	0.608 ^a^
No	105	19.1	105	19.1	210	19.1	
I do not know	58	10.5	37	6.7	95	8.6	
Frequency of dentist visits							
Once/year	37	6.7	5	0.9	42	3.8	0.005 ^b^*
Twice/year	27	4.9	84	15.3	110	10.0	
When is a problem	486	88.4	461	83.8	948	86.2	

^a^ Pearson chi-square test; ^b^ Fisher’s test; * *p* < 0.05.

**Table 3:** Children’s dental health indicators according to the daily consumption of dairy products.

Daily Consumption	Bistriţa-Năsăud Children (*n* = 275)	Mureş Children (*n* = 275)	Daily Consumption	Bistriţa-Năsăud Children (*n* = 275)	Mureş Children (*n* = 275)
	DMFT Mean ± SD	DMFT Mean ± SD		dmft Mean ± SD	dmft Mean ± SD
Milk			Milk		
Yes (*n* = 236)	1.86 ± 1.7	2.66 ± 2.4	Yes (*n* = 210)	6.20 ± 3.4	16.47 ± 12.4
No (*n* = 314)	2.20 ± 2.0	3.49 ± 3.4	No (*n* = 259)	6.53 ± 3.4	17.20 ± 12.4
*p*	0.273	0.087	*p*	0.567	0.888
Yogurt			Yogurt		
Yes (*n* = 160)	2.03 ± 1.9	2.48 ± 2.7	Yes (*n* = 137)	6.32 ± 3.7	14.79 ± 12.2
No (*n* = 390)	2.24 ± 1.9	3.23 ± 3.8	No (*n* = 332)	6.40 ± 3.2	16.16 ± 11.4
*p*	0.285	0.740	*p*	0.897	0.055
Cheese			Cheese		
Yes (*n* = 257)	1.95 ± 1.8	2.99 ± 2.7	Yes (*n* = 223)	6.30 ± 3.4	16.91 ± 12.3
No (*n* = 293)	2.17 ± 1.9	5.02 ± 4.9	No (*n* = 246)	6.36 ± 3.4	17.77 ± 12.5
*p*	0.413	0.036 *	*p*	0.908	0.474
Other dairy products			Other dairy products		
Yes (*n* = 84)	1.83 ± 1.6	2.61 ± 2.7	Yes (*n* = 76)	5.80 ± 3.4	14.29 ± 12.7
No (*n* = 466)	2.11 ± 1.9	3.23 ± 3.7	No (*n* = 393)	6.43 ± 3.5	17.91 ± 12.2
*p*	0.440	0.309		0.370	0.786

Independent sample *t* test; * *p* < 0.05.

**Table 4:** Children’s dental health indicators according to the daily consumption of sugary foods or added sugar.

Daily Consumption	Bistriţa-Năsăud Children (*n* = 275)	Mureş Children (*n* = 275)	Daily Consumption	Bistriţa-Năsăud Children (*n* = 275)	Mureş Children (*n* = 275)
	DMFT Mean ± SD	DMFT Mean ± SD		dmft Mean ± SD	dmft Mean ± SD
Sugar			Sugar		
Yes (*n* = 162)	2.29 ± 1.9	3.43 ± 2.4	Yes (*n* = 136)	6.35 ± 3.4	18.16 ± 13.6
No (*n* = 388)	1.98 ± 1.9	3.0 ± 1.6	No (*n* = 333)	6.28 ± 3.4	16.97 ± 11.8
*p*	0.288	0.432	*p*	0.897	0.566
Chocolate			Chocolate		
Yes (*n* = 55)	3.14 ± 1.8	5.43 ± 4.6	Yes (*n* = 50)	5.31 ± 2.9	14.99 ± 10.6
No (*n* = 495)	1.95 ± 1.9	2.88 ± 2.3	No (*n* = 419)	6.45 ± 3.5	17.60 ± 12.5
*p*	0.011 *	0.007 *	*p*	0.173	0.336
Sweets			Sweets		
Yes (*n* = 13)	3.65 ± 2.3	7.65 ± 7.3	Yes (*n* = 13)	5.25 ± 4.4	17.05 ± 16.5
No (*n* = 537)	2.0 ± 1.9	3.02 ± 2.4	No (*n* = 456)	6.36 ± 3.4	17.33 ± 12.3
*p*	0.066	0.009 *	*p*	0.477	0.976
Cookies			Cookies		
Yes (*n* = 31)	3.13 ± 2.1	4.38 ± 3.7	Yes (*n* = 24)	8.49 ± 2.9	24.71 ± 8.2
No (*n* = 519)	2.0 ± 1.9	3.06 ± 2.9	No (*n* = 445)	6.22 ± 3.4	16.93 ± 10.4
*p*	0.009 *	0.215		0.056	0.028 *

Independent sample *t* test; * *p* < 0.05.

**Table 5:** Prevalence of dental caries (dmft and DMFT) among school-age children, by county and age group.

Age Group	County	Total Number of Children	Children with dmft/DMFT > 0	Caries Prevalence (%)
6 years	Bistriţa-Năsăud	275	224	81.5%
6 years	Mureş	275	215	78.2%
10 years	Bistriţa-Năsăud	275	183	66.5%
10 years	Mureş	275	167	60.7%

**Table 6:** Correlation between the body composition measurements and dental health indicators.

	Dental Health Indicators							
	Group of Bistriţa-Năsăud Children (*n* = 275)		Group of Mureş Children (*n* = 275)		Group of Bistriţa-Năsăud Children (*n* = 275)		Group of Mureş Children (*n* = 275)	
Body Composition Measurement	DMFT		DMFT		dmft		dmft	
	r	*p*	r	*p*	r	*p*	r	*p*
Height [cm]	0.471	0.005 **	0.387	0.005 **	–0.407	0.005 **	–0.192	0.017 *
Weight [kg]	0.414	0.005 **	0.320	0.005 **	0.350	0.005 **	–0.154	0.048 *
Waist [cm]	0.247	0.006 **	0.194	0.014 *	–0.217	0.018 *	–0.122	0.180
The ratio of waist to hip	0.123	0.264	0.190	0.043 *	–0.036	0.729	–0.022	0.826
Body fat [%]	0.094	0.124	0.039	0.332	–0.090	0.325	–0.109	0.158
BMI [kg/m^2^]	0.228	0.007 **	0.161	0.045 *	–0.187	0.049 *	–0.130	0.322

* *p* < 0.05; ** *p* < 0.01.

**Table 7:** The logistic regression model predicting Romanian children’s dental caries from Bistriţa-Năsăud and Mureş counties.

Independent Variable	Crude Odds Ratio (COR)	*p*	Adjusted Odds Ratio (AOR)	*p*
Mother’s education level				
Primary level	3.45 (1.29, 9.39)	0.017 *	2.69 (0.82, 9.06)	0.013 *
Secondary level	1.81 (0.97, 3.61)	0.036 *	1.29 (0.51, 3.38)	0.078
Higher level	1			
Father’s education level				
Primary level	2.45 (0.51, 3.38)	0.022 *	1.42 (0.61, 3.37)	0.063
Secondary level	1.55 (0.65, 3.78)	0.073	1.22 (0.50, 3.10)	0.176
Higher level	1			
Frequency of daily brushing				
Once/day	1			
Twice/day	0.92 (0.48, 1.80)	0.498		
Irregular	1.32 (0.68, 2.65)	0.704		
Frequency of daily sugar intake				
Daily	1.66 (0.81, 3.46)	0.059	1.72 (0.85, 3.53)	0.032 *
Occasional	1			
Body fat ratio	1.13 (1.08, 1.26)	0.009 *	1.17 (1.09, 1.27)	0.008 *

* *p* < 0.05.

## Data Availability

All the raw data presented in this study can be provided upon request by the corresponding author.
